# Editorial: Driving extracellular vesicles toward applications in precision medicine

**DOI:** 10.3389/fmed.2022.1049697

**Published:** 2022-11-22

**Authors:** Somchai Chutipongtanate, Kovit Pattanapanyasat, Jarek Meller, Juana Serrano López

**Affiliations:** ^1^Division of Epidemiology, Department of Environmental and Public Health Sciences, University of Cincinnati College of Medicine, Cincinnati, OH, United States; ^2^Pediatric Translational Research Unit, Department of Pediatrics, Faculty of Medicine Ramathibodi Hospital, Mahidol University, Bangkok, Thailand; ^3^Department of Clinical Epidemiology and Biostatistics, Faculty of Medicine Ramathibodi Hospital, Mahidol University, Bangkok, Thailand; ^4^Chakri Naruebodindra Medical Institute, Faculty of Medicine Ramathibodi Hospital, Mahidol University, Samut Prakan, Thailand; ^5^Center of Excellence for Microparticle and Exosome in Diseases, Research Department, Faculty of Medicine Siriraj Hospital, Mahidol University, Bangkok, Thailand; ^6^Division of Biostatistics and Bioinformatics, Department of Environmental and Public Health Sciences, University of Cincinnati College of Medicine, Cincinnati, OH, United States; ^7^Department of Biomedical Informatics, University of Cincinnati College of Medicine, Cincinnati, OH, United States; ^8^Division of Biomedical Informatics, Cincinnati Children's Hospital Medical Center, Cincinnati, OH, United States; ^9^Experimental Hematology Laboratory, IIS Hospital Universitario Fundación Jiménez Díaz, Madrid, Spain

**Keywords:** biomarkers, clinical applications, drug carriers, exosomes, extracellular vesicles, microvesicles, precision medicine, therapeutic

Extracellular vesicles (EVs) have become a growing topic of discussion in precision medicine. EVs is a generic term for non-replicated, lipid bilayer encapsulated nano-scaled particles released from living cells (1). In a broad view, EVs have three subtypes, i.e., exosomes (small EVs), microvesicles (large EVs), and apoptotic bodies (apoptotic cell-derived EVs), which differ in biogenesis pathways, particle properties, and molecular markers (1). Depending on their cell hosts, EVs contain functional cargos, including proteins, nucleic acids, and lipids. In biology, EVs play crucial roles in cell-to-cell communication, maintaining cellular homeostasis, regulating tissue development, and modulating immune functions (1–5). Characterizing EV-derived molecules in biological and disease states would lead to the discovery of precise biomarkers. EVs share the cytoplasm of pathogenic cells and are present in extracellular fluids. For example, one can follow this concept to develop a liquid biopsy for detecting oncogene amplification status through EV-derived mRNA upregulation, e.g., MYCN-amplified neuroblastoma (2), in adjunct to the routine clinical workup for the risk classification and subsequent therapeutic regimens.

In this direction, Carnino et al., from the Boston University Medical Campus, United States, contributed an excellent review article covering EV biology and analytical procedures for EV study and summarized the current evidence of EV-derived miRNAs as the disease biomarkers and therapeutic targets of respiratory diseases, including asthma, chronic obstructive pulmonary disease, acute lung injury, and lung cancers. Given that EVs contain molecular signatures of the host cells, local biofluids, i.e., nasal, bronchoalveolar or pleural lavages and exhaled breath condensates serve as a unique source of EV-based liquid biopsy for precise diagnosis of inflammatory conditions and malignant respiratory diseases. Monnamorn et al., from the Prince of Songkla University, Thailand, investigated the diagnostic potential of plasma-derived large EVs (lEVs) for septic shock, a life-threatening condition caused by infectious etiologies. By longitudinal monitoring of the concentration of plasma-derived lEVs in 21 patients with septic shock vs. 9 with non-sepsis infection, the high plasma concentration of lEVs was associated with the onset of septic shock, and plasma lEVs concentrations gradually decreased over the course of patient recovery. By quantifying and subtyping lEVs, they found platelets, erythrocytes and endothelial cells, but not leukocytes, were the significant contributors of lEVs in both septic shock and non-sepsis-infected patients. While this result warrants further validations in a larger cohort, this study provides a logical approach to enumerating plasma lEV subtypes and quantities as part of biomarker discovery in complex diseases.

EVs contain functional molecules, e.g., surface ligands and receptors, cytosolic enzymes and signaling molecules, and nucleic acids, particularly miRNAs (1, 3). Identifying EV-derived miRNAs with validated target specificity would lead to miRNA-based targeted therapy. Harnessing the unique characteristics of original cell sources through their EVs would lead to advance therapeutic applications (3–5), for example, mesenchymal stem cell-derived EVs for stem cell-free therapy (4) and milk-derived EVs for drug carriers (5).

Along this line of EV research, Zhou et al., from the Fudan University, China, investigated milk-derived EVs as drug nanocarriers to overcome cisplatin-resistant ovarian cancer. They demonstrated that cisplatin-loaded milk EVs, but not cisplatin alone, can evade endosomal trapping, a major mechanism of cisplatin resistance, thereby enhancing the anti-cancer effect of cisplatin in cisplatin-resistant ovarian carcinoma *in vitro* and *in vivo*. Focusing on another promising source for EV-drug carriers, Chiangjong et al., from the Mahidol University, Thailand, comprehensively reviewed the current state of red blood cells as the source of an EV-based drug delivery system. Since EVs share plasma membranes with their origin cells, red blood cell-derived EVs (RBCEVs) are recognized as a relatively safe and biocompatible drug delivery system. They discussed the advantages and challenges of RBCEVs compared to conventional synthetic vehicles and non-RBC-derived EVs. A proposed strategy of RBCEV production from self-blood (autologous) or the blood group-matched packed red cell units (allogenic) and loaded with therapeutic agents (e.g., miRNAs or targeted inhibitors) warrants further investigation in precision medicine.

One of the most critical questions in EV research is how EVs migrate through different tissues and barriers to reach their recipient cells. Koomullil et al., from the University of Alabama at Birmingham, United States, proposed a simulation model of EV transport within the tumor microenvironment (TME) by considering the EV physical properties and concentration gradients, the TME physiological factors (such as interstitial flow and diffusion), and the cellular factors of EV production by donor cancer cells and EV uptake by the recipient immune cells. Then they validated this simulation by using a sophisticated EV transport model with 3D transwell geometry. This computational approach facilitates our understanding of cell-to-cell communication in the TME mediated through EVs and has potentially applicable in a broader context to determine therapeutic EV transport across various biological barriers and tissues toward recipient cells.

To this end, this Research Topic covers broad aspects of EV applications in the context of precision diagnosis and treatment. While this Research Topic includes articles that significantly contribute to the rise of EV applications for precision medicine, this research field is still in its infancy. Future investigations using a prospective multicenter cohort or population study would ensure the usefulness of EV-based liquid biopsy in general populations. Molecular characterization focusing on the surface ligands of different EV subtypes would improve our understanding of potential targeted tissues and recipient cells. Functional studies of both native and bioengineered EVs, together with investigating more efficient methods to load EV nanocarriers with the drugs of interest, would drive EVs toward therapeutic applications in precision medicine.

## Author contributions

SC wrote the first draft of the manuscript. KP, JM, and JS reviewed the manuscript and provided critical feedback. All authors read and approved the final version of the manuscript.

## Funding

SC was supported by the Office of National Higher Education Science Research and Innovation Policy Council of Thailand (PMU-B), Grant Numbers B17F640004 and B05F630082. KP was supported by the National Research Council of Thailand (NRCT), Grant Number N42A650870.

## Conflict of interest

The authors declare that the research was conducted in the absence of any commercial or financial relationships that could be construed as a potential conflict of interest.

## Publisher's note

All claims expressed in this article are solely those of the authors and do not necessarily represent those of their affiliated organizations, or those of the publisher, the editors and the reviewers. Any product that may be evaluated in this article, or claim that may be made by its manufacturer, is not guaranteed or endorsed by the publisher.
